# Genome-wide gene expression analysis in the amphioxus, *Branchiostoma belcheri* after poly (I: C) challenge using strand-specific RNA-seq

**DOI:** 10.18632/oncotarget.21553

**Published:** 2017-10-06

**Authors:** Qi-Lin Zhang, Zheng-Qing Xie, Ming-Zhong Liang, Bang Luo, Xiu-Qiang Wang, Jun-Yuan Chen

**Affiliations:** ^1^ LPS, Nanjing Institute of Geology and Paleontology, Chinese Academy of Science, Nanjing, China; ^2^ State Key Laboratory of Pharmaceutical Biotechnology, School of Life Science, Nanjing University, Nanjing, China; ^3^ Department of Marine Science, Qinzhou University, Qinzhou, China; ^4^ Guangxi Academy of Fishery Sciences, Nanning, China

**Keywords:** Branchiostoma belcheri, transcriptome analysis, strand-specific RNA-seq, poly (I:C), antiviral immunity, Immunology

## Abstract

The gene expression associated with immune response to bacteria/bacterial mimic has been extensively analyzed in amphioxus, but remains largely unknown about how gene are involved in the immune response to viral invasion at expression level. Here, we analyze the rRNA-depleted transcriptomes of *Branchiostoma belcheri* using strand-specific RNA-seq in response to the viral mimic, poly (I:C) (pIC). A total of 5,317 differentially expressed genes were detected at treatment group by comparing with control. The gene with the most significant expression changes (top 15) after pIC challenge and 7 immune-related categories involving 58 differently expressed genes were scrutinized. By functional enrichment analysis of differently expressed genes, gene ontology terms involving response to stress and stimulus, apoptosis, catabolic and metabolic processes and enzyme activity were overrepresented, and several pathways related to immune signaling, immune response, cancer, apoptosis, viral disease, metabolism were activated after pIC injection. A positive correlation between the qRT-PCR and strand-specific RNA-seq data confirmed the accuracy of the RNA-seq results. Additionally, the expression of genes encoding NLRC5, CASP1, CASP6, CYP450, CAT, and MDA5 were induced in *B. belcheri* under pIC challenge. Our experiments provide insight into the immune response of amphioxus to pIC and valuable gene expression information for studying the evolution of antiviral immunity in vertebrates.

## INTRODUCTION

Amphioxus, a living representative of the Cephalochordata, has been used extensively in research on the evolutionary and comparative immunology of vertebrates due to its primitive immune system among chordates and status as the closest living proxy of the vertebrate ancestor [1-3]. To date, the amphioxus *Branchiostoma belcheri* has been widely used as an experimental system among studies involving amphioxus immunity; with the *B. belcheri* genome sequence now determined [4], it has become an even more important and useful model animal for investigating the primitive immune response of vertebrates [5].

Previous studies on this topic have concentrated on exploring the immune response of amphioxus to various bacteria [6, 7], such as *Vibrio anguillarum*, *Staphylococcus aureus,* and *Escherichia coli*, and to non-viral mimics [5, 8], such as the Gram-negative bacterial lipopolysaccharide (LPS), lipoteichoic acid (LTA), and polyhydroxyalkanoates (PHA). However, few studies have focused on the antiviral response of amphioxus, and those reports that do exist are based on a small number of genes. For example, Liu et al. [9] discovered a RIG-I-like receptor in amphioxus, BjLGP2, and verified its antiviral function in *Branchiostoma japonicum* challenged by poly (I:C) (pIC). Similarly, Lei et al. [10] identified an acute response of a gene encoding an antiviral protein, viperin (BjVip), to pIC challenge and demonstrated viperin-mediated antiviral capacity in *B. japonicum*. Despite these studies, the global gene expression response of amphioxus to viral injection remains largely unclear.

High-throughput analysis (HTA) technologies have been widely used in investigating gene expression changes in amphioxus against non-viral challenge, especially LPS injection. We recently examined the immune responses of microRNAs (miRNAs) and identified some miRNAs with acute responses to LPS challenge in the gills of *B. belcheri* using microarray technology [5]. Liu et al. [11] identified several key immune signaling genes in LPS-challenged amphioxus using expressed sequence tags (ESTs). Thus, many genes with acute immune responses and biomarkers have been identified using cDNA microarray, ESTs, and suppression subtractive hybridization (SSH) [12] in amphioxus over the last decade. However, there have been virtually no reports involving HTA technology directly relevant to the immune response against viruses or viral mimics in amphioxus. Recently, strand-specific cDNA sequencing technology (ssRNA-seq) has emerged as a tool that provides more comprehensive and integrated sequence information by analyzing full transcript sequences. It allows for direct acquisition of information on the originating strand, accurately demarcates boundaries between adjacent genes, and reveals accurate expression levels of overlapping transcripts [13]. Additionally, analysis of ribosomal RNA (rRNA)-depleted libraries allows for the detection of genes with low expression much more efficiently, as mRNAs are fully retained after the removal of rRNA; conversely, enrichment of mRNA with polyA tails using magnetic beads with polyT leads to a certain amount of transcript loss [14, 15]. Now, transcriptomic analysis of rRNA-depleted libraries using ssRNA-seq has been widely used to compare the expression profiles of long non-coding RNAs (lncRNAs) and mRNAs in mammals [16, 17], fish [18], and insects [19]. Thus far, however, no researchers have used this approach to analyze gene expression changes in amphioxus.

Here, we injected pIC, a double-stranded RNA (dsRNA) viral mimic, into healthy adult *B. belcheri*. Next, we identified differentially expressed genes (DEGs) between control animals and those of pIC treatment by analyzing ssRNA-seq data; moreover, we performed functional annotation of DEGs and those with critical roles in immune response and the most different expression (top 15) were obtained. Then, functional gene ontology (GO) term and Kyoto Encyclopedia of Genes and Genomes (KEGG) pathway enrichment analysis of DEGs were performed. Finally, we selected a group of DEGs to validate the ssRNA-seq results and surveyed expression variation among them involving immune signaling, detoxication and apoptosis in a replicated experiment using quantitative real-time PCR (qRT-PCR). This study contributes to the further investigation of the evolution of antiviral mechanisms in vertebrates.

## RESULTS

### Genome-wide identification of DEGs

RNA samples at various time points after pIC challenge were pooled together prior to sequencing, which may lead to bias in the results (e.g. a gene may first be up-regulated and down-regulated subsequently); however, we aimed at obtaining an overview of what occurs during immune challenge rather than understanding of dynamic changes of gene expression. The control and treatment libraries were sequenced, and we obtained a total of 94.56 million and 94.63 million clean reads, respectively, after filtering the raw reads in each library. Only 0.04% and 0.03% of clean reads in the control and treatment groups, respectively, were matched to rRNA sequences using a bioinformatics approach before they were filtered out, indicating that the majority of the rRNA was removed effectively by the reagent kits. Of the remaining clean reads, 46.53% and 45.75% of control and treatment reads, respectively, were mapped to protein-coding gene loci. The unique merged transcript set contained 30,388 sequences, including 23,812 (78.35%) and 24,388 (80.24%) expressed genes (reads per kilobase per million reads, RPKM value > 0.1) in the control and treatment groups, respectively, which were used for further analysis. A total of 5,317 DEGs were identified between control *B. belcheri* and those treated with pIC for 24 h. By comparing the treatment group to controls, 2,938 and 2,379 DEGs were determined to be up- and down-regulated, respectively, following pIC injection (Supplementary Table 1). Table 1 presents the top 15 most up- and down-regulated DEGs in immune-challenged *B. belcheri*. The most up-regulated gene encodes leukocyte cell-derived chemotaxin 1(LECT1); in addition, three genes encoding histones exhibited up-regulated expression. Conversely, a gene encoding chondroitin proteoglycan 2 (CPG2) exhibited the most down-regulated expression. Down-regulated genes also included some well-known sequences encoding tumor necrosis factor receptor superfamily member 16 (NGFR), adhesion G protein-coupled receptor B2 (ADGRB2), complement component C7 (C7), and interferon-induced protein 44 (IFI44). A few genes were annotated to hypothetical proteins, and their functions were therefore unknown.

**Table 1:** Top 15 most up- and down-regulated genes between control and treatment groups.

GeneID	log2Ratio(treatment/control)	Description	Gene name	FDR
**The top 15 most up-regulated genes**				
001470R	13.78	Leukocyte cell-derived chemotaxin 1	LECT1	0
161330F	12.38	Hypothetical protein	/	7.99E-06
141980F	12.05	EGF-like domain-containing protein 1	SCUBE1	7.73E-244
024370R	11.86	Histone H1	H1	1.73E-220
250030R	11.40	Lecithin retinol acyltransferase	LRAT	3.59E-175
141990F	10.64	C-type lectin 6	CTL6	1.03E-116
101420F	10.53	RNA-directed DNA polymerase from mobile element jockey	POL	2.79E-110
277890F	10.53	Hypothetical protein	/	3.65E-110
084230F	10.45	Synapse differentiation-inducing gene protein 1-like	SYNDIG11	3.29E-105
024270F	10.36	Histone H2B	H2B	1.49E-100
024300F	10.34	Histone H2A	H2A	1.42E-99
276140F	10.01	Receptor-transporting protein 3	RTP3	2.74E-83
209870F	10.00	Mucin-like protein 1	MUCL1	4.98E-83
261040F	9.60	FAS-associated death domain protein	FADD	6.14E-67
094460R	9.36	Natural killer cells antigen CD94	KLRD1	8.78E-59
**The top 15 most down-regulated genes**				
146060R	-13.19	Chondroitin proteoglycan 2	CPG2	8.81E-123
112420F	-12.65	Tumor necrosis factor receptor superfamily member 16	TNFRSF16	0.000131
074740F	-12.47	Type I inositol 3,4-bisphosphate 4-phosphatase	INPP4A	1.60E-11
096490F	-12.42	Hypothetical protein	/	1.43E-303
180950F	-12.09	Latent-transforming growth factor beta-binding protein 1	LTBP1	3.19E-257
050170F	-11.88	Adhesion G protein-coupled receptor	AGPCR	3.90E-231
073740R	-10.76	Uromodulin	UMOD	1.11E-128
157820R	-10.75	Guanylate-binding protein 5	GBP5	1.27E-27
073640F	-10.71	Protein eva-1 homolog C	EVA1C	2.20E-125
093180R	-10.53	Delta-like protein 4	DLL4	4.37E-227
050100R	-10.38	Complement component C7	C7	6.05E-210
254530F	-10.07	E3 ubiquitin-protein ligase MIB1	MIB1	0
009460R	-9.89	Hypothetical protein	/	7.56E-81
115020F	-9.66	Epidermal growth factor-like protein 7	EGFL7	1.18E-71
115020F	-9.66	Interferon-induced protein 44	IFI44	1.18E-71

### Analysis of DEGs associated with immune responses

The results showed that 58 DEGs involving immune response had been identified under pIC challenge (Table 2), including 33 up-regulated and 25 down-regulated DEGs. Previous studies have conducted a comprehensive genomic survey of immune gene repertoires in amphioxus genome, *Branchiostoma floridae* [20]. Based on catalogs/cluster of immune genes in *B. floridae* and grass carp (*Ctenopharyngodon idellus*) [20, 21], we grouped these DEGs into 7 catalogs, including heat shock proteins (HSPs), complement and coagulation cascades, pattern recognition receptors (PRRs), cell adhesion, cytokines, apoptosis, adaptors and signal transducers and caspases.

**Table 2:** DEGs typically involved in immune responses.

Category and gene name	log2Ratio	Diff	FDR	Gene name
**Heat shock proteins**				
Heat shock 70 kDa protein	4.64	UP	0.000346	HSP70
Heat shock factor protein 5	3.11	UP	8.48E-06	HSF5
Small heat shock protein 25	2.24	UP	2.43E-31	HSP25
Activator of 90 kDa heat shock protein ATPase homolog 2	-1.21	DOWN	1.63E-11	AHSA2
Heat shock protein 83	-1.73	DOWN	0	HSP83
Heat shock protein HSP 90	-2.27	DOWN	0	HSP90
Heat shock 22 kDa protein	-2.93	DOWN	2.38E-85	HSP22
**Complement and coagulation cascades**				
Coagulation factor V	6.18	UP	1.02E-09	F5
Fibrinogen gamma chain	4.25	UP	1.96E-13	FGG
Coagulation factor VII	2.15	UP	7.02E-07	F7
Alpha-tectorin-like	2.12	UP	4.79E-09	TECTA
Urokinase-type plasminogen activator	1.35	UP	6.42E-14	PLAU
Coagulation factor XII	1.16	UP	2.69E-07	F12
Haptoglobin-like	-1.56	DOWN	1.99E-05	HP
Coagulation factor IX	-1.84	DOWN	3.38E-06	F9
Complement component C1q-like	-2.87	DOWN	1.17E-09	C1qL
Complement component C8 beta	-8.90	DOWN	0	C8B
Complement component C7	-10.38	DOWN	6.05E-210	C7
**Pattern recognition receptors (PRRs)**				
C-type lectin 6	10.64	UP	1.03E-116	CTL6
Protein NLRC5	5.96	UP	1.44E-08	NLRC5
Toll-like receptor 1	5.05	UP	3.38E-05	TLR1
NACHT, LRR and PYD domains-containing protein 9	2.25	UP	6.95E-75	NLRP9
NACHT, LRR and PYD domains-containing protein 4	2.01	UP	1.46E-19	NLRP4
NACHT, LRR and PYD domains-containing protein 3	1.66	UP	7.00E-21	NLRP3
Soluble scavenger receptor cysteine-rich domain-containing protein	-1.02	DOWN	1.10E-53	SRCR5D
C-type lectin 9a	-1.50	DOWN	8.06E-07	CTL9A
Peptidoglycan-recognition protein (PGRP) SD	-2.06	DOWN	1.44E-45	PGRP-SD
Peptidoglycan-recognition protein (PGRP) SC2	-2.09	DOWN	4.15E-54	PGRP-SC2
Melanoma differentiation associated genec5	-2.22	DOWN	7.07E-19	MDA5
Intelectin-1	-2.60	DOWN	4.75E-19	ITLN1
Scavenger receptor cysteine-rich domain-containing protein	-3.27	DOWN	1.90E-22	SRCR4D
**Cell adhesion**				
Cadherin-like protein 23-like	2.22	UP	2.86E-25	CDH23L
Integrin beta-4	-1.04	DOWN	3.77E-79	ITGB4
Claudin-11	-2.26	DOWN	1.02E-19	CLDN11
**Cytokine**				
Interleukin-17D	2.69	UP	1.35E-13	IL17D
Tumor necrosis factor alpha	1.82	UP	4.90E-16	TNFα
Interferon regulatory factor 6	1.35	UP	7.11E-31	IRF6
Interleukin 17 receptor D	1.11	UP	0.000949	IL17DR
Inhibin beta E chain	-2.38	DOWN	6.26E-55	INHBE
Interferon-induced protein 44	-9.66	DOWN	1.18E-71	IFI44
**Apoptosis**				
FAS-associated death domain protein	9.60	UP	6.14E-67	FADD
Apoptosis inhibitor IAP	2.65	UP	1.05E-201	IAP
cAMP-dependent protein kinase	2.07	UP	2.37E-15	PKA
Caspase activity and apoptosis inhibitor 1	1.32	UP	1.31E-08	CAAP1
Apoptosis-inducing factor 2	-3.23	DOWN	1.14E-58	AIFM2
**Adaptors, signal transducers and caspases**				
Apoptotic initiator caspases (Caspase-2)	9.20	UP	9.60E-54	CASP2
Tumor necrosis factor receptor superfamily member 6B	8.55	UP	1.82E-37	TNFRSF6
Caspase-1	6.24	UP	1.18E-18	CASP1
Apoptotic initiator caspases (Caspase-8)	4.75	UP	0.000192	CASP8
TNF receptor-associated factor 2	4.19	UP	1.45E-66	TRAF2
Apoptotic effetor caspases (Caspase-3)	1.70	UP	1.59E-07	CASP3
Apoptotic effetor caspases (Caspase-7)	1.51	UP	1.99E-18	CASP7
TNF receptor-associated factor 3	1.19	UP	1.80E-44	TRAF3
TNF receptor-associated factor 1	-1.02	DOWN	2.69E-07	TRAF1
TNF receptor-associated factor 6	-2.62	DOWN	2.12E-162	TRAF6
Myeloid differentiation primary response protein MyD88	-3.36	DOWN	3.40E-07	MyD88
Apoptotic effetor caspases (Caspase-6)	-6.62	DOWN	2.37E-24	CASP6
Tumor necrosis factor receptor superfamily member 16	-12.65	DOWN	0.000131	TNFRSF16

### GO and KEGG enrichment analysis of DEGs

All DEGs were matched to the three GO subgroups to further analyze their functions. The results showed that genes that were differentially expressed in response to pIC challenged were significantly enriched in 106 GO terms (the false discovery rate, FDR < 0.01), including 46 (43.40%) biological process terms, 16 (15.10%) cellular component terms, and 44 (41.51%) molecular function terms (Supplementary Table 2). For the biological process category, GO terms involved in metabolic processes, apoptosis, response to stimulus, and catabolic processes were commonly overrepresented. Among the cellular component category, the top three GO terms were cell (GO:0005623), cell part (GO:0044464), and intracellular (GO:0005622) based on their significance level in descending order. Most GO terms belonging to the molecular function subgroup were associated with nucleotides, ion binding, activity of metabolic enzymes, and so on. Moreover, we noted GO terms clearly involved in immune response in the biological process subgroup, such as response to stress (GO:0006950), response to chemical stimulus (GO:0042221), response to stimulus (GO:0050896), and so on. Interestingly, we detected 14 GO terms directly-related to immune response in the biological process (marked by asterisk in the Supplementary Table 2), such as response to stress (GO:0006950), response to chemical stimulus (GO:0042221), response to stimulus (GO:0050896), and so on, and those associated with apoptosis, including cell death (GO: 0008219), death (GO:0016265), programmed cell death (GO:0012501), and apoptotic process (GO:0006915).

In the KEGG enrichment analysis, we detected a total of 25 significantly enriched (FDR < 0.01) pathways (Table 3). Among these pathways, 11 pathways were related to cancer and human diseases, including viral myocarditis (ko05416), arrhythmogenic right ventricular cardiomyopathy (ARVC) (ko05412), prostate cancer (ko05215), and so on. Notably, the list of enriched KEGG pathways also presented many pathways directly related to antiviral immune response in *B. belcheri* (marked by pound sign in Table 3), including NOD-like receptor signaling pathway (ko04621), cytokine-cytokine receptor interaction (ko04060), MAPK signaling pathway (ko04010), RIG-I-like receptor signaling pathway (ko04622), NF-κB signaling pathway (ko04064), Toll-like receptor signaling pathway(ko04620), antigen processing and presentation (ko04612), and one apoptosis-related pathway, apoptosis (ko04210).

**Table 3:** Results of pathway enrichment analysis of DEGs, sorted by significance levels.

Pathway ID	Pathway	FDR
ko05416	Viral myocarditis	5.09E-06
ko05412	Arrhythmogenic right ventricular cardiomyopathy (ARVC)	7.53E-06
ko04621	#NOD-like receptor signaling pathway	2.11E-05
ko05215	Prostate cancer	4.74E-05
ko05200	Pathways in cancer	1.08E-04
ko04060	#Cytokine-cytokine receptor interaction	1.14E-04
ko05410	Hypertrophic cardiomyopathy (HCM)	5.27E-04
ko05133	Pertussis	5.30E-04
ko00590	Arachidonic acid metabolism	7.10E-04
ko01100	Metabolic pathways	8.12E-04
ko05218	Melanoma	8.92E-04
ko05320	Autoimmune thyroid disease	8.92E-04
ko04370	VEGF signaling pathway	2.95E-03
ko05322	Systemic lupus erythematosus	2.95E-03
ko05414	Dilated cardiomyopathy	3.01E-03
ko00071	Fatty acid metabolism	3.01E-03
ko04010	#MAPK signaling pathway	3.19E-03
ko00592	alpha-Linolenic acid metabolism	3.19E-03
ko04210	#Apoptosis	4.06E-03
ko04622	#RIG-I-like receptor signaling pathway	4.57E-03
ko00350	Tyrosine metabolism	5.06E-03
ko05134	Legionellosis	7.37E-03
ko04064	#NF-kappa B signaling pathway	8.22E-03
ko04620	#Toll-like receptor signaling pathway	9.16E-03
ko04612	#Antigen processing and presentation	4.89E-02

#: Directly immune-related KEGG pathways

### Validation of ssRNA-seq results and survey of gene expression using qRT-PCR

**Figure 1:**
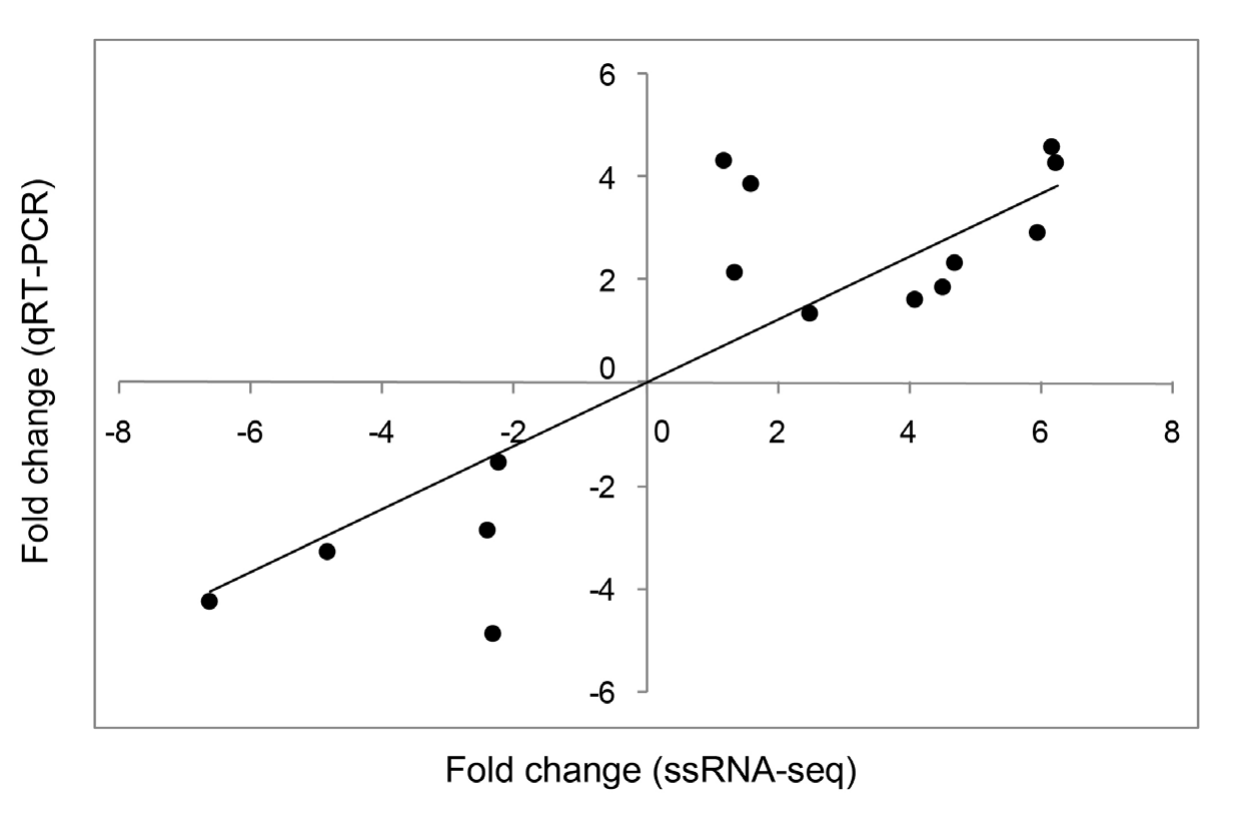
Correlation between relative fold changes in expression based on ssRNA-seq and qRT-PCR analyses 15 differentially expressed genes (DEGs) identified by ssRNA-seq were assessed by qRT-PCR of the same samples. Each black dot indicates the relative expression fold change between the treatment and control groups according to ssRNA-seq (x-axis) and qRT-PCR (y-axis). IBM SPSS Statistics 22 was used for statistical analysis.

**Figure 2:**
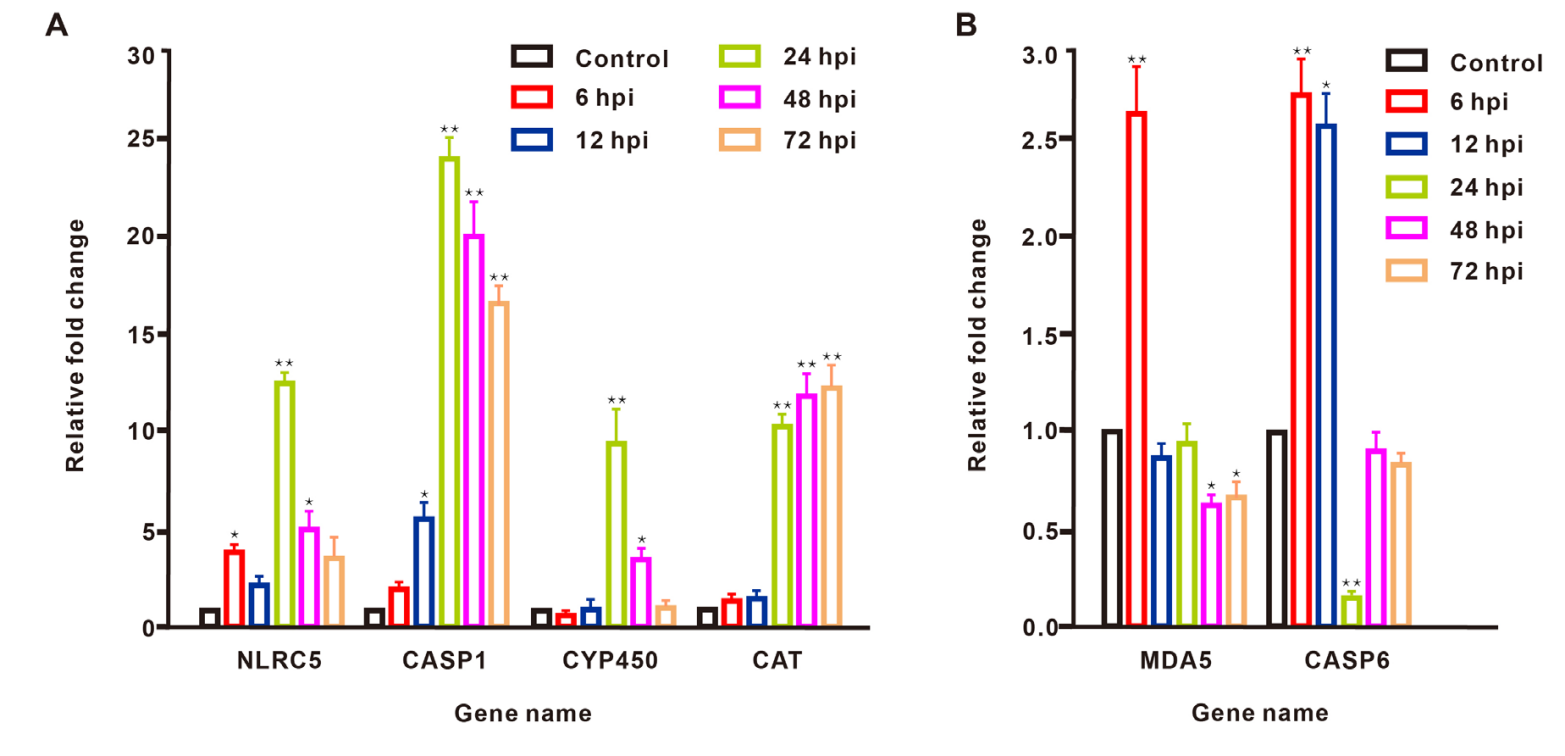
Dynamic expression changes of six DEGs in *B. belcheri* injected with pIC and assessed at different time points The results are presented as relative to the expression level of *EF1A* and are shown as the mean± SD. *P < 0.05 and **P < 0.01 vs. control. IBM SPSS Statistics 22 was used for statistical analysis, and significance was evaluated by one-way ANOVA test.

Correlation analysis of the fold changes in gene expression ratios between the ssRNA-seq and qRT-PCR data showed a highly significant positive correlation (Pearson’s correlation coefficient, *r* = 0.878, *P* < 0.001; Figure 1). Therefore, the qRT-PCR analysis supported the accuracy of the ssRNA-seq methods in this study. In the survey of gene expression, our results showed that the expression level of *NLRC5* was significantly up-regulated at 6, 24, and 48 hour-post injection (hpi) compared with levels in the control group and reached a peak at 24 hpi. The expression level of *CASP1* was significantly elevated at 12, 24, 48, and 72 hpi, reaching a peak at 24 hpi. The expression level of *CYP450* was significantly up-regulated only at 24 and 48 hpi. The expression profile of *CAT* showed a continuous increase in expression over 72 hpi, including significant up-regulation at 24, 48, and 72 hpi. *MDA5* was significantly up-regulated at 6 hpi, but its expression fell below the level in controls at 12, 24, 48, and 72 hpi. The expression level of *CASP6* was significantly up-regulated at 6 and12 hpi, showing the highest expression at 6 hpi, but significantly decreased at 24 hpi, after which the expression level fell below that in controls at 48 and 72 hpi (Figure 2).

## DISCUSSION

Compared with other conventional methods of cDNA library preparation, such as polyA enrichment, more genes with lower expression levels and slightly more accurate gene expression levels can be detected in rRNA-depleted libraries [15]. In addition, rRNA-depleted libraries are useful for retaining not only protein-coding genes but also lncRNAs and small nucleolar RNAs (snoRNAs) [18, 19, 22]. Indeed, in this study, in addition to the ∼45% of clean reads that could be mapped to protein-coding gene loci, we identified ∼10,000 non-coding transcripts using the remaining clean reads and bioinformatics tools, including ∼4,000 lncRNAs (unpublished, data not shown). These identified non-coding RNAs should be further verified by experiments, especially the lncRNAs, as there are no previous reports on lncRNAs in amphioxus. In addition, Zheng et al. [22] demonstrated that ssRNA-seq could be used to generate more reliable gene expression results than conventional library preparation in many cases. In this study, a strong positive correlation indicated agreement between the ssRNA-seq and qRT-PCR results of 15 DEGs; thus, analysis of rRNA-depleted libraries using ssRNA technology was able to rapidly, fully, and accurately detect gene expression levels during large-scale expression profile analysis of amphioxus under pIC challenge conditions.

Here, we identified several significantly enriched GO and KEGG terms involved in organic acid and amino acid metabolism and catalysis after pIC treatment in amphioxus. This was also reported in previous studies, in which the spleen and caecum of Chinese Langshan chickens were challenged by pIC [23]. Yang et al. [24] reported that hepatitis B virus injection altered expression of lipid metabolism genes in transgenic mice by regulating oxidative stress and interacting with regulatory proteins associated with organic acid metabolism. The amount of amino acids needed for protein synthesis decreases when cells are damaged by viral infection, but some cells recruit more amino acids to participate in antiviral immunity [25], which may explain a few of the enriched GO terms involved in amino acid metabolism in this study. Therefore, we propose that alterations in organic acid and amino acid metabolism caused by viral invasion may represent an ancient response pattern in the antiviral immunity of vertebrates, especially in liver cells, as a similar phenomenon is reported frequently following hepatovirus injection in mammals [24, 25].

Because it lacks an adaptive immune system, amphioxus must strongly rely on innate immunity as a first line of defense line following immune stimulation [26, 27]. The most significant expression change (top 15) was observed in some well-known genes encoding proteins involved in innate immune response, such as complement component C7, mucin-like protein 1 [28], interferon-induced protein, adhesion G protein-coupled receptor [29], and natural killer cell antigen CD94; these DEGs could be useful molecular immune indicators in cephalochordates under virus challenge. Through RNA-seq analysis, recent studies have shown that genes belonging to the antigen processing and presentation pathway were overrepresented among DEGs identified in duck spleen infected with enteritis virus [30]. Here, the antigen processing and presentation pathway was included in the list of significant KEGG terms, suggesting that this pathway plays a key role in the antiviral immunity of amphioxus. Besides, cytokine-cytokine receptor interaction and MAPK signaling pathway identified in this study were also reported in pathway enrichment for DEGs in spleen of *Schizothorax prenanti* after pIC challenge [31]. As reported in previous studies, most genes involving stimulus and stress responses were also related to immune response [21, 26], thus it is worthwhile to note that some DEGs and enriched GO and KEGG terms were related to stimulus and stress responses. In biological processes involved in response to stimulus and stress, some well-known genes were identified, such as those encoding CYP450 and CAT. CYP450 function has been demonstrated in insects, where it is primarily involved in insecticide resistance and response to stimulus [32]. H_2_O_2_ overexpression is induced by pathogen infection in the clam *Meretrix meretrix*, which then up-regulates CAT to avoid oxidative damage and maintain the effectiveness of the immune system by eliminating excess H_2_O_2_ [33]. Our qRT-PCR analysis showed up-regulation of *CYP450* and *CAT* genes, especially in later immune stages within 72 hpi, indicating that pIC challenge likely triggered oxygen toxicity in amphioxus. These findings suggested that antiviral immune response is innate complexity and comprehensiveness in amphioxus.

To further investigate the mechanism of effect on innate immune response of amphioxus by pIC challenge, 58 directly immune-related DEGs are categorized into 7 categories. Key members identified in the current study in these categories and their functions in the context of antiviral immune responses are discussed in detail below. 1) HSPs are molecular chaperones that enhance the development of immune responses against virus infection [34]. Previous studies have showed that HSP70, HSP90 and small HSPs (sHSPs) were induced in response to virus challenge [34, 35]. In mammals, HSP70 could activate natural killer cells and induce innate immunity by the secretion of immune inductive cytokines [36, 37]. Oladiran et al. also reported the macrophages and pro-inflammatory cytokines were activated by recombinant HSP70 in goldfish (*Carassius auratus*) [38]. Our research detected that different expression of HSPs and their activator was induced by pIC challenge. It could be due to the HSPs simultaneously enhanced proliferation of immune cells and activity of cytokines [34]. In amphioxus, only HSP70 gene was cloned, and then its effectively thermo-inducible function was identified [39], thus the research on the immune function of HSPs is largely unknown. In the future, HSP molecule types and their complicate regulatory networks need to be elucidated in amphioxus. 2) The complement is an essential system for recognizing pathogens, immune surveillance in innate immune defense [20]. The complement could be initiated by one or a combination of three pathways, the alternative, lectin and classical. C1q is an essential activator for the classical complement pathway [21]. Huang et al. speculated that C1q-like (C1qL) may has function as lectin by biochemistry experiments and homologous comparison of function with lamprey C1q in *B. floridae*, while also found that this protein could inhibit platelet agglutination similarly to the mammalian C1q function in *B. japonicum* [20]. In this study, we found that complement component *C1qL*, *C7*, *C8b* of *B. belcheri* exhibited a down-regulated expression after pIC challenge, indicating the inhibition of lectin and classical complement pathways. We speculated that classical system may be an original complement pathway in chordates. 3) PRRs mainly express in innate immune cell and direct recognition to the pathogen molecules. The PRRs of vertebrates now are grouped into four primary groups as follows: RIG-l-like receptors (RLRs), NOD-like receptors (NLRs), Toll-like receptors (TLRs), and C-type lectin receptors (CLRs) [21, 40]. NLRC5, NLRP 3, 4, 9 identified in our list of DEGs, several member of the NLR family, are PRRs and key intracellular sensor in the innate immune response. The gene encoding NLRC5 was the most different expression in RNA-seq results, and then our qRT-PCR analysis identified that expression of this gene was significantly induced by pIC. Furthermore, the NLRs are known to inhibit the NF-κB signaling pathway by activating an inhibitor of nuclear factor kappa-B kinase (IKK) subunit in antiviral immunity [41]; both the NLR and NF-κB signaling pathways were enriched in our study. Additionally, MDA5, a member of the RLR family, acts as an innate sensor for recognizing dsRNA viruses. MDA5-mediated antiviral immunity relies on functionally active LGP2 [42]. Interestingly, an amphioxus LGP2 protein, BjLGP2, was identified and found to have antiviral activity following pIC challenge in *B. japonicum* [9]. In this study, the identified DEGs were significantly enriched for genes involved in the RLR signaling pathway; besides, *MDA5* showed an acute expression change to pIC challenge. Yuan et al. [43] reported that the expression level of *TLR1* (the only TLR identified to date in amphioxus) was suppressed in the digestive system of *B. belcheri* injected with pIC; our KEGG enrichment analysis of DEGs also implicated the TLR signaling pathway. However, up-regulated expression of *TLR1* was detected in our results. This inconsistency may be partially explained by the different sample type used in respective experiments. The *CTL6* and *CTL9A* exhibited in the list of DEGs, two C-type lectin that acts as a key PRR in innate immunity [26]. Chen et al. [44] reported that C-type lectin receptors (CLRs) interact with the leukocyte cell-derived chemotaxin 2 (LECT2) of *Plecoglossus altivelis* in the immune response, and expression of *LECT2* was induced by Singapore grouper iridovirus in *Epinephelus coioides* [45]. Here, the *LECT1* was the most significantly up-regulated among all DEGs, suggesting that the LECT gene family may be pivotal in the antiviral response in aquatic animals. The genes belonging to all the four main PRR groups presented significant immune response to pIC challenge, indicating that, similar to vertebrates, amphioxus has evolved a complex and diverse PRR system against viral invasion. 4) Cytokines consist of chemokines, interferons, interleukins and tumor necrosis factors (TNFs), and so on, which are generated by activated immune-related cells and regulated progress of immune responses [21]. In the current study, chemokines and interferons were not detected according to our analysis. Similarly, interferons, most interleukins, chemokines were not identified in sequenced *B. floridae* genome by systematically genomic analysis of the immune genes [20]. Moreover, the non-vertebrate type of some immune genes was employed in amphioxus. For example, two structural types of TLRs were identified in amphioxus, including the vertebrate-like TLRs and the protostome-like TLRs [20]. Probably, there was invertebrate-specific chemokines and interferons in amphioxus, but vertebrate-like type may not be existed. Interleukin-17 (IL-17), including IL17A-F members, generally secreted by Th17 cells specifically boosts a pro-inflammatory response after pIC challenge by directly synergizing with antiviral signaling in human cells, and IL-17-poly(I:C) synergy must rely on the presence of the interferon regulatory factor (IRF) [46]. We found that IL-17D, IL-17D receptor, IRF6 were presented in the list of DEGs, implying primitive members of IL-17 family. Besides that, tumor necrosis factor alpha (TNFα) could initiate higher cytokine and chemokine secretion in human epithelial cells infected by influenza A virus [47], thus up-regulation of the gene encoding TNFα in our results would lead to production of cytokine and chemokine. 5) In adaptors, signal transducers and caspases, tumor necrosis factor receptor superfamily members (TNFRSFs). The biological functions of TNFRSFs encompass beneficial effects for the host in immune and defense response [48]. Furthermore, the TNF receptor-associated factor (TRAF) family could activate NF-kappa B and MAPK signaling pathway by linking receptors of Toll and TNFRSFs to signaling cascades [49]. In this study, many DEGs encoding TNFRSFs and TRAF were identified, and NF-kappa B and MAPK signaling pathway were significantly enriched, suggesting that this signaling cascade have been informed in cephalochordates. 6) The activation of adhesion molecules could promote recovery of injury caused by pathogen invasion [21]. We firstly revealed that genes encoding adhesion molecules participated in antiviral immunity in amphioxus using RNA-seq. 7) Apoptosis is a process of programmed cell death that removes excess, injured, and potentially threatening cells [26], and virus-induced apoptosis occurs when cells are invaded with a virus, leading to cell death [50]. Here, DEGs were enriched to some GO categories and pathway associated with cell apoptotic processes. Moreover, some DEGs involving apoptosis were seen significantly induced as well, particularly those genes encoding apoptotic caspases (Caspase-1, 2, 3, 6, 7, 8). Caspase-1 cleaves gasdermin D (GSDMD) and then directly or via signaling cascade pathway leads to lytic apoptosis [51]. Caspase-6, a downstream enzyme in the caspase-mediated cascade, is often processed by caspase-3 or is self-activated in the absence of other caspase members, playing an important function in apoptosis and inflammation signaling pathways [52]. Our results showed that the expression levels of genes encoding caspase-1 and caspase-6 gradually declined following pIC challenge, presenting a significant down-regulation at 24 hpi. Mutations in uromodulin (UMOD), a protein abundant in mammalian urine, can induce cellular apoptosis [53], and a gene encoding this protein was identified among the top 15 most down-regulated genes. FAS-associated death domain protein (FADD), an adaptor protein for members of the tumor necrosis factor receptor superfamily (TNFRSF), induces apoptosis in mammalian cells after transfection *in vitro* [54]. The expression change of *FADD* and *TNFRSF16* were significantly induced by pIC challenge. This finding reveals that a complex and diverse network involving cell apoptosis exists in *B. belcheri*, suggesting that apoptosis may be a key biological process for antiviral immune response in amphioxus.

Histones can be clearly distinguished into five major families, including H2A, H2B, H3, and H4, known as the core histones, and their linker counterparts (H1/H5), which play a key role in antimicrobial peptide activity in vertebrates, antiviral immune processes in mammals, and control of chromatin-related DNA damage and transcription [55, 56]. Moreover, histone ubiquitination is important for many regulatory processes involved in gene transcription and duplication, such as DNA repair and transcript elongation, thereby triggering gene activity [57]. A member of the histone H2A family was identified in *Macrobrachium rosenbergii* and found to be significantly up-regulated following white spot syndrome viral challenge [58]. Valero et al. [55] found that expression of genes encoding histone H1 and H2B was significantly up-regulated in the brain and head-kidney, respectively, of *Dicentrarchus labrax* treated with nodavirus. Previous studies have demonstrated that a fish virus-induced protein exhibited an E3 ubiquitin-protein ligase function and could regulate antiviral signaling by ubiquitination [59]. Combined with our data on the response of genes encoding histones and ubiquitin ligase to pIC injection in the present study, we speculate that histone ubiquitination-activated immune-related genes protect amphioxus from viral infection.

Notably, we identified many enriched pathways associated with diseases, including viral myocardiopathy, pertussis, autoimmune disease, and cancer, in this study. It is known that hallmarks of disease caused by defects in basic signaling pathways and gene mutations can more or less be found in ancient animals [26, 60]. Srivastava et al. [60] reported that many genes participate in human disease and cancer according to comparative genomic analysis of the sponge *Amphimedon queenslandica.* In addition, researchers can easily find many congeners of genes that related to human genetic diseases in *Drosophila melanogaster*, thus instigating a database collection of human disease gene cognates in *Drosophila* that was constructed over 15 years ago [61]. Our analysis shows that amphioxus not only possesses functional genes linked to human disease but also initiates its antiviral response by expressing many genes involved in human disease-related pathways. Therefore, amphioxus may be a favorable system for investigating the origin of genetic diseases in vertebrates.

In conclusion, in our experiment, RNA-seq was used to explore antiviral immune responses in *B. belcheri* infected with pIC for the first time. We identified many DEGs, GO terms, and KEGG pathways associated with immune response, apoptosis, diseases, metabolism, and detoxification. Our findings reveal a complex and diverse network of immune responses against pIC challenge in amphioxus, and demonstrate that the antiviral immune system in amphioxus shares certain similarities to that in vertebrates. Moreover, identification of some novel genes with acute immune responses will allow for a deeper understanding of mechanisms of antiviral immunity in basal chordates. These results provide useful information for tracing the evolution of antiviral immunity in vertebrates. Although the functional relevance of some genes in the present study could not be explained due to the complexity of the immune system, these may become clear in future studies.

## MATERIALS AND METHODS

### pIC injection and sample collection

We obtained *B. belcheri* adults from Beihai Marine Station of Nanjing University (Beihai City, Guangxi Province, China). The animal breeding method was described in our previous studies [5, 62]. To empty the contents of the amphioxus digestive systems, they were starved for a week in filtered seawater at 24–28°C. Only obviously healthy amphioxus specimens were used for further experiments. Next, approximate 30 adult individuals with nearly consistent size (4.5±0.3 cm) were averagely divided into two groups (15 individuals in each group), including treatment and control. Then, pIC dissolved in PBS (100 μg/ml final concentration, 15 μl/individual) was injected into the enterocoelia (intraperitoneal injection) of each individual in the treatment group, and an equal dose of PBS was injected into control according to previously described methods [5, 62]. Subsequently, we randomly selected four amphioxus individuals from the treatment groups at 6, 12, 24 hpi. Simultaneously, we collected samples in PBS control at the same time point with pIC treatment groups.

### RNA extraction, library construction, and sequencing

Total RNA was extracted from whole body of each individual using TRIzol reagent (Invitrogen, USA) following the manufacturer’s protocol. Residual genomic DNA was digested by RNase-free DNase (Qiagen, Germany). RNA concentration and purity were measured by a NanoDrop ND1000 spectrophotometer (Thermo Scientific, USA). RNA structural integrity and quality were verified by Agilent 2100 Bioanalyzer (Agilent Technologies, USA). RNA was pooled equally from the 12 samples in each group for RNA sequencing and therefore temporal expression data was lost. For construction of strand-specific libraries, we first removed rRNA using Ribo-Zero rRNA Removal Kit (Epicenter, USA). Next, the purified rRNA-depleted samples were fragmented using fragmentation buffer. Finally, we constructed strand-specific sequencing libraries using TruSeq Stranded Total RNA Sample Preparation Kits (Illumina, USA). The prepared libraries were sequenced on a HiSeq 2000 Sequenator (Illumina, USA) by the Beijing Genomics Institute (BGI, China).

### Filtering, mapping, and assembly of sequences

We performed quality control of the raw data using the FastQC suite (http://www.bioinformatics.bbsrc.ac.uk/projects/fastqc/). Raw reads were trimmed and filtered using SOAPnuke (https://github.com/BGI-flexlab/SOAPnuke) to discard adapter sequences, invalid reads with unknown bases, or low-quality sequences (>50% bases with *Q* value ≤ 10). Filtered cleans reads were mapped to public rRNA databases (http://togodb.biosciencedbc.jp/togodb/view/frnadb_summary#en) using SOAP2 (version 2.2.1, http://soap.genomics.org.cn/), and these mapped reads were removed. Next, all high-quality, clean reads were mapped to the *B. belcheri* reference genome (v18h27.r3; http://genome.bucm.edu.cn/lancelet/) using the sliced read mapper TopHat (version 2.1.0; http://ccb.jhu.edu/software/tophat/index.shtml). Reads mapped by TopHat were used to assemble transcripts with Cufflinks (version 2.02) [63]. A unique transcript set was produced by merging all assembled transcripts using Cuffmerge [64]. All transcripts were searched against the NCBI non-redundant protein sequence (Nr), Universal Protein Resource (UniProt) (http://www.uniprot.org/), GO (http://www.geneontology.org/), and KEGG (http://www.genome.jp/kegg) databases using NCBI BLAST to obtain gene functional information. The expression level of each gene in each library/group was then calculated using Cufflinks based on RPKM values [63].

### Identification and analysis of DEGs

Genes with an RPKM value < 0.1 were filtered out, and the remainder were retained as expressed genes. DEGs between controls and the pIC-treated group were detected using DEGseq in the R package (http://www.bioconductor.org/). To control FDR of multiple pairwise comparisons, the significance levels of all candidate DEGs were corrected with the Benjamini and Hochberg (BH) methods [65]. Genes with an absolute value of the fold change (FC) of gene expression ≥ 2 (|log_2_ ratio| ≥ 1) and an FDR < 0.001 were considered DEGs. To further understand the functional classification of DEGs and altered pathways in amphioxus following injection with pIC and to gain useful information for exploring potential differences in gene expression between control and treated amphioxus groups, we conducted GO and KEGG enrichment analysis using the Blast2GO pipeline [66] and KOBAS software (http://kobas.cbi.pku.edu.cn), respectively. The significance levels of GO terms and KEGG pathways were corrected by FDR. We used a rigorous significance criterion (FDR < 0.01) to identify enriched targets.

### Validation of ssRNA-seq data and detection of gene expression using qRT-PCR

We selected 15 DEGs (10 up- and five down-regulated DEGs) to validate the ssRNA-seq data using qRT-PCR. pIC injection, sample collection, total RNA extraction and sample pooling was performed according to above mentioned methods in ssRNA-seq. Samples with three biological replications were collected at each timing points, and then three control vs. treatment groups were generated as three biological replications. Subsequently, to survey the dynamic variation in gene expression in *B. belcheri* challenged with pIC at the different time points, we selected six key immune-related DEGs involving different function from 15 DEGs. These six DEGs were well-known members involving immune cell surface receptors, apoptosis signaling cascades and detoxification in antiviral immunity, including NLR family CARD domain containing 5 (*NLRC5*), melanoma differentiation-associated protein5 (*MDA5*), caspase 6 (*CASP6*), caspase 1 (*CASP1*), cytochrome P450 (*CYP450*), and catalase (*CAT*), and they were investigated in greater detail in a replicated experiment using *B. belcheri* challenged with pIC for 6, 12, 24, 48, and 72 h time points. Experimental sampling and RNA purification were conducted according to the above-mentioned methods of sample collection and RNA extraction. Beacon Designer 7 was used to design specific primer pairs used in qRT-PCR (Supplementary Table 3). qRT-PCR was performed on the ABI 7300 Real-Time PCR System (Applied Biosystems, USA) using SYBR Premix ExTaq (Takara, Japan) using the technological processes and reaction systems used in our previous studies [62]. *EF1A* was used as a reference gene, and relative gene expression was calculated using the 2^-∆∆CT^ method [67, 68, 69]. qRT-PCR reaction of each gene for each assay was included three technical replications and three biological replications, and final normalized results in expression survey of six genes are presented as means ± standard deviation (SD).

### Data accessibility

The data supporting the results of this article have been submitted to NCBI Sequence Read Archive (SRA) repository [Experiment accession numbers: SRX2610969].

## SUPPLEMENTARY MATERIALS TABLES






