# Supplementary material for: Genome-wide gene expression analysis in the amphioxus, Branchiostoma belcheri after poly (I: C) challenge using strand-specific RNA-seq
Source: Oncotarget. 2017 Oct 6;8(65):108392–405. doi: 10.18632/oncotarget.21553 (PMC5752451; doi:10.18632/oncotarget.21553)
Supplement: Supplementary file 3 [file oncotarget-08-108392-s003.docx]

**Supplementary Table 3: Primers used in quantitative RT-PCR.**

| Gene symble | ID | Sequence (5'-3') | Size (bp) |
| --- | --- | --- | --- |
| *IL17D* | 067000R | F: ATTTGCTGTCGCCCTCAT | 109 |
|  |  | R: TCTGCTCTGTCTCCTTCTCT |  |
| *INHBE* | 174290F | F: CACCACACCTCCGTCCTA | 137 |
|  |  | R: AAGATGTAGCCGTCGTTGTC |  |
| *IRF6* | 193550R | F: TCCTCACCGTCCACAGAT | 77 |
|  |  | R: TCACATCCTTCATCCAGACTTG |  |
| *NLRC5* | 095820R | F: CACTCGCTACACTCGATAGG | 96 |
|  |  | R: CTCCAGGCTGAGGTCATTC |  |
| *MDA5* | 128740R | F: ACCTGCTTGACTGGATACG | 133 |
|  |  | R: CTGATGACCTCTCGCTGTT |  |
| *CASP6* | 024310F | F: AGAGCAGGCTGGAGAAGA | 142 |
|  |  | R: TTTGGCATTGAACGGCTTTAG |  |
| *CASP1* | 164550F | F: GTGAATCGGAAGCCAAGAGA | 123 |
|  |  | R: CTCTCCTCCTCAGTTCTTCTTC |  |
| *CYP450* | 184520R | F: GGTGCGTGAATCCGAGAT | 108 |
|  |  | R: TGTTCTTGCCTAGCGACTC |  |
| *CAT* | 218780F | F: GGTCCTGAGCAGAATGAGTC | 82 |
|  |  | R: CCTCGTCAGCAGTGTTGT |  |
| *ARSJ* | 264710F | F: GGTATCAGATTCACTATGG | 86 |
|  |  | R: GGGTAGGGTAATTTCATC |  |
| *ACPP* | 057650R | F: CTGCCGAGGAAGATGTGTT | 146 |
|  |  | R: TCCTGTCCGTAGAGTCAAGT |  |
| *KLHL26* | 257430F | F: ACTCAGAAGCAGGTGTTGG | 122 |
|  |  | R: TATTGTCGGTGGTTGGAAGG |  |
| *CLU1* | 266280F | F: GCTGCTATGTGGAAGAAGAGAT | 147 |
|  |  | R: TTCGCTGGTGGACAATCG |  |
| *F2* | 046760F | F: GGGAGGCACACAACAACA | 86 |
|  |  | R: TTGAAGTCAGGATGGACGATT |  |
| *ANGPTL4* | 056930F | F: CGGGTTGTCGTCGTTATCT | 130 |
|  |  | R: TGCCATCTCACCTGAATCAC |  |
| *EF1A* | 210670 F | F: CTGTGCCGTGCTGATTGTA | 145 |
|  |  | R: GGTGGAGTCCATCTTGTTGAC |  |
